# Evidence of Pervasive Legacy of Land Use Change on Dung Beetles in Central European Grazed Grasslands

**DOI:** 10.1002/ece3.73571

**Published:** 2026-04-29

**Authors:** Elisabeth Glatzhofer, Bernd Lenzner, Tobias Schernhammer, Franz Essl

**Affiliations:** ^1^ Vienna Institute for Nature Conservation and Analyses Vienna Austria; ^2^ Division of BioInvasions, Global Change & Macroecology, Department of Botany and Biodiversity Research University of Vienna Vienna Austria

**Keywords:** extinction debt, insect decline, livestock farming, pasture ecosystems, Scarabaeoidea, time lags

## Abstract

Over the last century, intensification and conversion of land use has caused habitat destruction and species extinctions throughout Europe. However, these extinctions can be delayed by time lag effects, i.e., extinction debts, which are typically measured by species richness. The aim of this study was to investigate whether such extinction debts can be detected in dung beetles of the Pannonian region of Austria and the Czech Republic, where extensive pasture systems were converted into intensive agricultural areas. We modeled the impact of this land use change and the use of anthelmintics as an additional stressor on both dung beetle species richness and individual abundance. We found that the use of veterinary anthelmintics significantly reduces dung beetle species richness and that historic land cover data from the 19th century best explained not only current dung beetle species richness, but showed even stronger effects on individual abundance. This suggest that population declines may precede species extinctions, potentially masking the true extent of biodiversity loss in short‐term studies. Biodiversity assessments using only species richness may therefore underestimate ongoing species losses, as they don't account for temporal trends in population size. Our findings further underscore the necessity of incorporating historical land‐use data when assessing biodiversity trends and conservation strategies, as well as the need for long‐term ecological monitoring to capture delayed responses to environmental change. Moreover, the observed impact of anthelmintics on dung beetle communities calls for reconsideration of veterinary practices to mitigate their unintended consequences on biodiversity. Overall, the results add a crucial temporal dimension to understanding insect declines, reinforcing that past land‐use decisions shape present and future biodiversity in complex and often underestimated ways.

## Graphical abstract

This study modeled the effects of livestock farming and land use change on dung beetle species richness and abundance in Austria and the Czech Republic. Using historic land cover data from the 19th century, we found evidence for extinction debt, showing that past landscapes better explained current dung beetle populations than recent land use. These findings highlight the importance of incorporating historical land‐use data and long‐term ecological monitoring in biodiversity assessments.

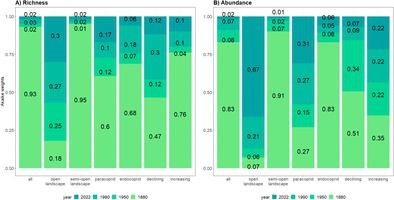

## 1 Introduction

In the past century, Central European agricultural systems and land use practices underwent profound changes, following the mechanization and intensification of the agricultural sector, and resulting in a transition from extensive, site‐specific agricultural practices toward intensive, industrial agriculture (Dallimer et al. 2009; Poschlod 2017; Cegielska et al. 2018). Particularly, extensive grazing systems and traditional agropastoral activities have been affected by this transformation (Poschlod 2017; Sartorello et al. 2020). The decline of extensive pasture farming was especially pronounced in lowlands such as the Pannonian region (Griffiths et al. 2013; Varga et al. 2016; Biró et al. 2018; Cegielska et al. 2018). Lowland pastures were either converted to arable land or afforested, leading to a homogenization of the landscape with stricter segregation of different land use types and the loss of habitat‐rich transitional zones (Kozak 2003; Otero et al. 2015; Poschlod 2017). Thus, many species dependent on open grassland habitats are suffering severe declines in Europe: These have been reported especially within insects such as beetles, hymenoptera, and butterflies, including species that provide important benefits for humans such as pollinators, natural pest enemies, and nutrient recyclers (Sánchez‐Bayo and Wyckhuys 2019), but also for grassland plant richness (Wesche et al. 2012).

One of the groups strongly affected by land‐use change are dung beetles, or coprophagous Scarabaeoidea (Sánchez‐Bayo and Wyckhuys 2019; Lumaret et al. 2022; Ruiz et al. 2025). They depend on dung as a resource for feeding, habitat and reproduction and are hence directly affected by declining numbers of grazing animals and pastures in the landscape (Jay‐Robert et al. 2008; Noriega et al. 2023). Dung beetles have also been shown to react especially sensitive to anthropogenic landscape modifications (Fattorini 2011; Filgueiras et al. 2015; Noriega et al. 2021) and loss of habitat heterogeneity (López‐Bedoya et al. 2022). This led to declines in dung beetle richness and diversity, particularly of highly specialized species, e.g., ball‐rolling species, habitat specialists, and selective feeders (Hutton and Giller 2003; Buse et al. 2015; Schernhammer et al. 2023). The increased prophylactic use of veterinary medicines on grazing animals since the 1990s, especially of anthelmintics (medicine administered against worm infestation), further accelerates dung beetle diversity declines (Tovar et al. 2023). This has far‐reaching consequences for the functioning of grazing ecosystems, as dung beetles provide essential ecosystem services: By removing and burying dung they substantially support soil aeration, water availability and nutrient cycling, and enhancing plant growth (Brown et al. 2010; Manning et al. 2016; Milotić et al. 2019) and providing more potential forage area for grazing animals (Nichols et al. 2008). They also promote secondary seed dispersal, and suppress pests by minimalizing potential reinfection areas (Nichols et al. 2008; Gregory et al. 2015; Milotic et al. 2017).

Species and populations responses to these declines and degradation of open landscape habitats vary depending on their life history traits and adaptive capacity (Scherreiks et al. 2022). Some might respond with a considerable time lag, resulting in extinction debt phenomena, where the extinction of species occurs with a certain time after the actual alterations, which causes a state where the species richness of a habitat patch is not in equilibrium with current environmental conditions (Tilman et al. 1994). Extinction debt has been documented particularly in long‐lived and less mobile taxa such as plants (Vellend et al. 2006; Gustavsson et al. 2007), whereas evidence for short‐lived insect groups remains mixed, with extinction debt reported for butterflies, hoverflies, beetles, and spiders in some studies (Sang et al. 2010; Bommarco et al. 2014; Scherreiks et al. 2022).

The aim of this study is to assess whether time‐lag effects of land‐use change are also detectable in dung beetles in the Austrian Pannonian region, being an area in which both pasture habitats and dung beetle diversity have declined continuously over the last decades (Schernhammer et al. 2023). We hypothesize that dung ‐beetle species richness and individual abundance are better explained by historical land‐use variables than by recent ones, indicating the presence of extinction debt. In addition, we aimed to investigate possible cumulative or interactive effects of habitat change and the use of veterinary anthelmintics on dung beetles, addressing a major knowledge gap, as existing studies have largely focused on the effects of either land‐use change or anthelmintics.

## 2 Material and Methods

### 2.1 Study Area and Sampled Pastures

**FIGURE 1:**
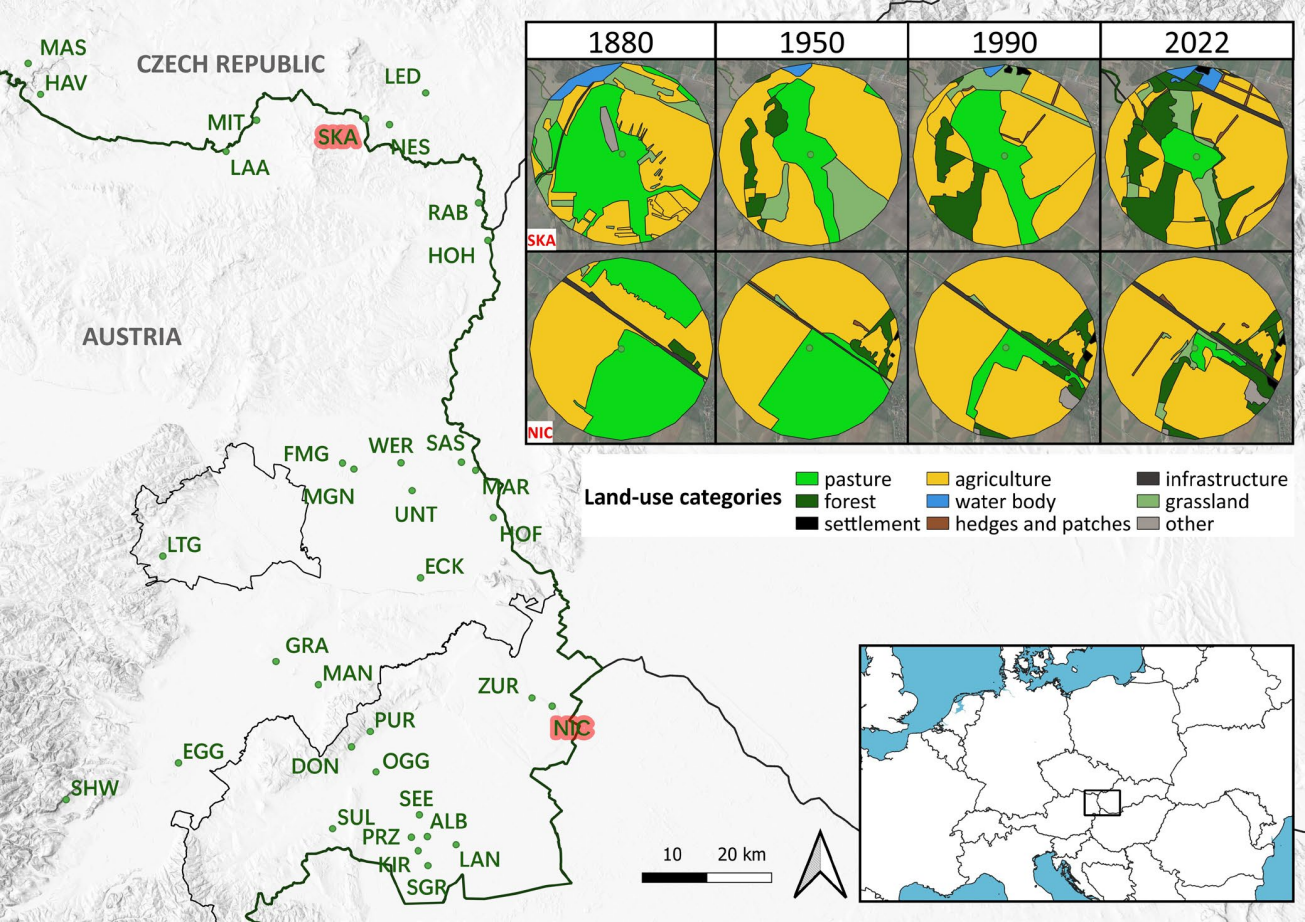
Study area and digitized land use change. The study area with the sampled pastures and two examples of land use change within 1 km radius over the four analyzed points in time, illustrating the decrease of pastures in the study region.

The study area comprised pastures in the Pannonian region of Eastern Austria (29 sites) and the adjacent Czech Republic (5 sites; Figure 1). The pastures varied in size from 1.2 ha to 860 ha, and sites were grazed by cattle or horses (Table S1). The climate in the study region is continental temperate with cool winters and warm summers, annual average temperature of 8°C–10°C, and an annual precipitation of 500–700 mm. The selection of pastures was based on farmers agreeing to participate in the study, grazing species (only cattle and horses were used; see Table S1), and best possible even spatial distribution. Sampling of dung beetles (coprophagous Scarabaeoidea) was carried out by collecting five dung pats as a random sample for each pasture. The pats were approximately the same age (24 h) and size and were excavated using a shovel to dig out the pats including 15 cm of soil beneath them. They were then transferred into a thick plastic bag, transferred into a plastic tub, and dung beetles therein sorted out using a floating method (Denver Museum of Nature and Science 2007). The specimens were then preserved in 70% alcohol. All pastures were sampled once between April and June on fine days (sunshine, 9:00 am–1:00 pm). 13 study sites were sampled in 2022; data from the remaining pastures was collected in previous investigations between 2018 and 2021 using the same sampling method (Table S1; Ambrožová et al. 2021; Schernhammer et al. 2023). In addition to the sampling, pasture owners were asked whether they used anthelmintics on the pasture or not (Table S1).

### 2.2 Species Identification and Ecological Traits

Species were identified using relevant literature (e.g., Krell and Fery 1992; Bunalski 1999; Rößner 2022), all specimens are located in the collection of the first author. For the subsequent calculation of the models, the collected beetle species were grouped differently according to their ecological features (hereinafter referred to as “ecological groups”), resulting in four different classifications by nesting behavior, habitat preferences, dung specialization, and changes of occurrence range within the study region. The data used for nesting behavior, habitat preferences and dung specialization was derived from Buse et al. (2018), who list these traits for all central European dung beetle species. Regarding nesting behavior, the majority of the species grouped into paracoprid (tunnelers) and endocoprid (dwellers) species. Four species showing other nesting behavior were not included in the models using this trait as they concerned only a few species—one dung roller and three phytosaprophagous species developing in detritus—and the latter are not considered to be direct dung decomposers (see Table S2). The separation into habitat preferences included species affiliated to open habitats only (hereinafter referred to as “open landscape species”) and species using semi‐open and/or forest habitats (hereinafter referred to as “semi‐open landscape species”; Table S2). For the species' degree of specialization (which in Buse et al. (2018) is estimated by how many different types of animal dung are used as a food source), low numbers indicate a high specialization. As all collected species except of one are polyphagous (feeding on at least three different kinds of animal dung; Table S2), no models with this trait were calculated. Lastly, species occurrence range was classified as stable, declining, or increasing following a species catalog from the study area (Schernhammer et al. 2023) and were calculated as the proportion of currently (2020) versus historically (before 1950) occupied grid cells. Values bigger than one indicate species with an increasing range (for simplicity hereinafter referred to as “increasing species”) and smaller values species with a declining range (hereinafter referred to as “declining species”; Table S2).

### 2.3 Current and Historic Land‐Cover Data

To assess the role of habitat availability in the wider landscape, we extracted and digitized land cover from historical maps and aerial photographs in a circular area of 1‐km around the center of the sampled pastures (conducted in QGIS Geographic Information System, version 3.28.2‐Firenze). We used historical maps and aerial photographs from three different time points representing distinct periods of major changes in agricultural practices: (i) c. 1880–1900, (ii) c. 1950–1960, (iii) c. 1990–1995 and current aerial photographs (2020–2022) for reference (information on maps and data sources is given in Table S5).

Nine land‐cover categories were considered: grasslands (ungrazed), pastures, forests, arable fields (incl. vineyards), water bodies, shrubland (e.g., hedges, patches of trees), settlements, infrastructure (e.g., roads, railways) and other (e.g., landfills, quarries) (Figure 1). For analysis, only the main categories pastures, arable fields, settlements and forests were used as predictors (representing the total area of each category within the 1‐km radius around each pasture center), as these are the categories that either provide suitable habitats for dung beetles (pastures), influence their occurrence the most (arable fields and forests as indicator for land use change and intensification and settlements as indicator for urbanization) and have the largest shares.

### 2.4 Mixed Models

To compare impacts of agricultural practices and changing land use we built Generalized Linear Mixed Models (GLMMs) with a Poisson error distribution using the “lme4” package (Bates et al. 2015) in the programming language R (Version 4.1.0; R Development Core Team 2022). To avoid collinearity across predictor variables in the models we checked predictor correlations using the ggpairs and the ggcorr function in the R package “GGally” (Schloerke et al. 2024). All predictor variable correlations were below 0.7, indicating a sufficiently low correlation to be used in the same model (Dormann et al. 2013). As pastures were sampled within a time span of three different months (Table S1) they included both typical spring‐ and summer‐species with different phenologies. In order to account for these differences in phenology and nevertheless enable a comparison of the samples, the sampling month was included in the GLMMs as a random effect. Since some sample points cluster together geographically (see Figure 1) an Autocorrelation Function plot was calculated for a generalized linear model with the same variables to preclude a possible bias from spatial autocorrelation (Venables and Ripley 2002). The result did not show any spatial autocorrelation bias (Figure S1). For comparison of the GLMM‐models we calculated the corrected Akaike information criterion (AICc), which contains a second‐order bias correction for small sample sizes, and McFadden's *R* ^2^ values (McFadden 1979). The models were then compared by means of Akaike weights based on the AICc (Burnham et al. 2004).

To examine the influence of anthelmintics compared to land use, we built GLMMs using landcover data from 2022 and treatment with anthelmintics (*yes* or *no*) as predictor variables. These models were built separately from the historic land cover models, as anthelmintic use is only common since the 1990s and information on the extent of use was only available from farmers currently administering the pastures. We built separate models for each ecological group (i.e., paracoprid and endocoprid, declining and decreasing and open landscape and semi‐open landscape species) and all species together, using the respective species richness as response variables (Table S4). The same models using only landcover data of 2022 (without anthelmintics) were used as null models to test for the actual impact of anthelmintic use.

For analysis of historical land use imprints, we built a series of separate GLMMs using the digitized land‐cover data (i.e., area of pastures, fields, settlements and forests in a circle of 1 km around the center of the pasture; see Figure 1) from the four distinct time periods (1880–1900, 1950–1960, 1990–1995, and 2020–2022) as predictor variables. As response variables, we used species richness and individual abundance in separate models, calculated (i) for all recorded species combined and (ii) separately for each of the six ecological groups. For each combination of land‐cover of the four time periods, species richness or individual abundance and ecological groups a separate model was fitted, resulting in a total of 56 models (Table S6).

**FIGURE 2:**
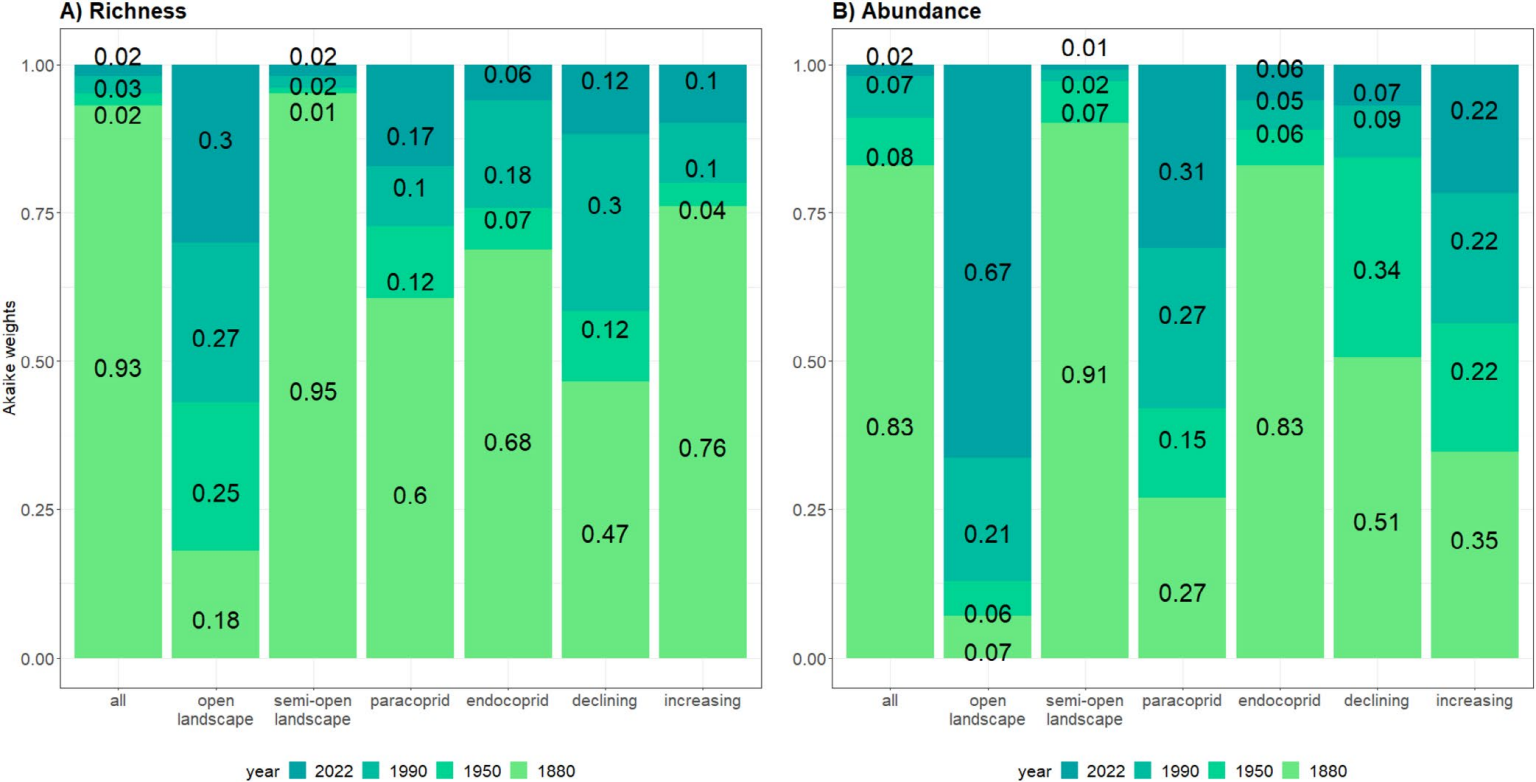
Akaike Weights for general linear mixed models. Relative support (Akaike Weights) for general linear models explaining the proportions of species richness (A) and abundance (B) of ecological groups by land use data of four different points in time. Diagnostic model values for each model (*p*‐values and estimates) can be found in Table S6.

To identify which land cover model best explained current species richness and individual abundance, we performed a model selection procedure based on the Akaike weights derived from the AICc (Figure 2; Dullinger et al. 2013).

## 3 Results

### 3.1 Dung Beetle Sampling

We collected 9738 beetles belonging to 56 species (Table S2). Species number per site varied between two and 24, with individual numbers ranging between three and 1491. Neither species nor individual numbers showed any dependence on pasture size (Pearson correlation coefficient of −0.15 (species richness, *p* = 0.4122) and −0.08 (individual richness, *p* = 0.6548)). Sixteen species exclusively use open habitats and were therefore assigned to the group of open landscape species, whereas 40 species are defined as semi‐open landscape species. Thirty‐two species are endocoprid (dwellers), 20 species paracoprid (tunnelers), and four show other nesting behaviors (phytosaprophagous species with development in detritus or telecoprids). The species occurrence trends according to Schernhammer et al. (2023) show that for the sampled species 16 species have been increasing since 1900, three are stable, and 37 species are declining in the study region.

### 3.2 Changes of Land Use

Between 1880 and 2022, the settlement and forest areas increased in all sample sites, while pasture area decreased sharply: Only six sites showed a clear increase in pasture size, of which two, however, are large‐scale intensive farms and for two others the exact change of pasture borders was not clearly visible from satellite images due to floodings. Change in agricultural arable fields varied, with an increase at 22 sites and a decrease at 12 sites (total land cover change is shown in Table S3).

### 3.3 Time Lags Caused by Land Use Changes

The model comparisons based on Akaike weights show that the model using land cover data from 1880 within a perimeter of 1 km around the sampling site clearly provided the best explanation for both total species richness and individual abundance (Figure 2, Table S6). A similar picture emerges for the models for the different ecological groups: Across four out of six ecological groups, historic land use data from 1880 explain species richness better than more recent ones; only species numbers of open landscape and paracoprid species were explained best by the newest land cover model. Similarly for individual abundance, the models using historic land cover data provide the best explanation and perform even better in most groups, also for open landscape and paracoprid species.

The influence of different landcover types varied strongly between the ecological groups (Table S6). Forest cover had a significant positive influence on semi‐open landscape species richness in earlier models, but this positive influence continuously decreases with newer models and is not significant in the 2022‐model. Conversely, for open landscape species richness, the impact of forest cover is significantly negative in the current landscape. Settlement areas had a significant negative influence on individual abundance of all groups over all time periods, with especially pronounced effects on endocoprid species, and emerged as the main driver for the occurrence of declining species in recent models. All model results and diagnostic values (*p*‐values and estimates) are given in Table S6 in the Supporting Information.

### 3.4 The Effects of Anthelmintics Application on Species Richness

**FIGURE 3:**
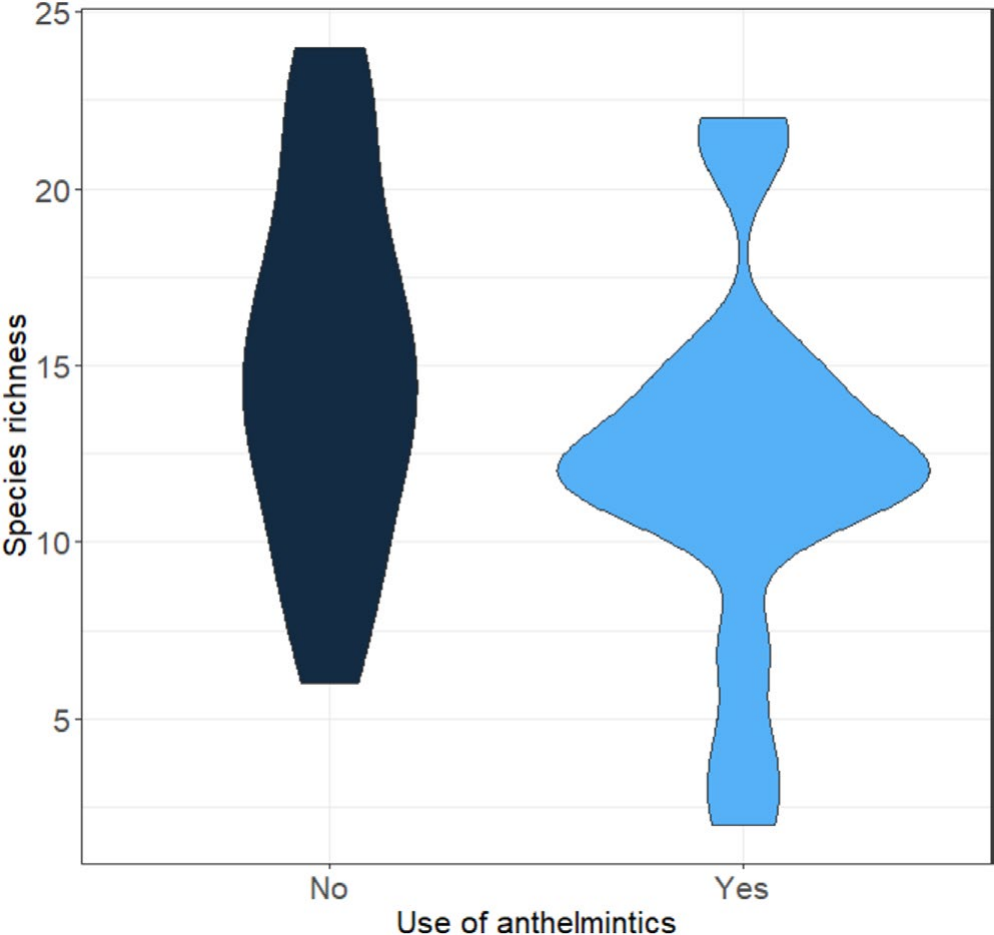
The impact of anthelmintics on species richness. Species richness on sampled pastures with (“Yes”) and without (“No”) use of anthelmintics.

The use of anthelmintics had a significant negative effect on overall species richness (Figure 3, Table S4). When compared to the GLMM without anthelmintics as an explanatory variable (null model using only land use data from 2022), the models including anthelmintics described species richness significantly better. The different ecological groups were affected by anthelmintics to a varying extent: while their application did not have a significant influence on paracoprid species, it had a strong significant negative impact on endocoprid species. They also seemed to affect semi‐open landscape species more than open land species. For increasing species only, anthelmintics were the only significant predictor, showing a strong negative impact; an effect that is also visible for declining species, albeit much weaker. All model results and diagnostic values (*p*‐values and estimates) are given in Table S4 in the Supporting Information.

## 4 Discussion

Our analysis demonstrates that land cover history in the surrounding landscape of the study sites has profound and long‐lasting impacts on current dung beetle communities, with detectable legacy effects spanning more than 130 years. For insects, some previous studies detected extinction debts (Bommarco et al. 2014; Löffler et al. 2020; Scherreiks et al. 2022), while others didn't find any effects of historic land use (Krauss et al. 2010; Deák et al. 2021). To our knowledge, this is the first study to explicitly test for extinction debt dynamics in dung beetles and, crucially, to disentangle time‐lagged responses of both species richness and individual abundance. Our results reveal that time lags are even more pronounced for individual abundance than for species richness, providing novel empirical support for the hypothesis that insect communities may persist for extended periods in a demographically eroded state before species losses become apparent (Tilman et al. 1994; Vellend et al. 2006). Such cryptic population declines are largely overlooked in extinction debt research, yet they are highly relevant given recent reports of dramatic reductions in insect biomass and abundance across Europe (Seibold et al. 2019; Ghisbain et al. 2024).

We further found that distinct ecological groups of dung beetles with different ecological preferences and levels of specialization differ in their lag times. For instance, forest cover, which expanded from 1880 to 2022 by 47.5%, positively affected semi‐open landscape species in the earlier 20th century, which shows that limited forest areas likely acted as refugia for these species within a landscape prevalently dominated by open grasslands. Conversely, open landscape dung beetles were negatively affected by increasing forest cover in recent history (Table S6).

In addition, we found that the widespread current use of anthelmintics in the study region negatively impacts dung beetle diversity, superimposing a novel cause of threat on dung beetles (Figure 3, Table S4), on top of the habitat degradation. These results support previous studies demonstrating the adverse effects of veterinary medicine on dung beetles (Krauss et al. 2010; Gregory et al. 2015; Milotic et al. 2017; Ambrožová et al. 2021). Treatment was shown to result in more homogenous dung beetle species communities with rare specialists disappearing first and generalist species remaining (Tonelli et al. 2017; Ambrožová et al. 2022), a pattern that was also visible in this study. Additionally, we observed varying effects of anthelmintics on species with different nesting behaviors: Endocoprid species experienced negative impacts, whereas paracoprid species remained unaffected. Some previous studies align with these results (Kless and Scholtz 2001), others, however, reported that paracoprid species were more adversely impacted (Sands and Wall 2018; Ambrožová et al. 2022; Tovar et al. 2023). The stronger effects on endocoprid species could be explained by the dung beetle larvae's increased sensitivity to medical substances compared to adults (Lumaret et al. 2012); and as the larvae of endocoprid species develop directly inside the feces, they are exposed to higher concentrations of medical substances. However, this hypothesis should be tested in further studies.

To conclude, our results provide evidence that the full extent of local insect species decline due to profound changes in land use may only be fully realized over extensive time periods. This has severe implications for conservation assessments as extinction risks and the full rate of species and population losses until equilibrium is reached may currently be strongly underestimated. Further, the importance of habitat loss as a driver of insect decline may be partially masked. By demonstrating that historical land‐use change leaves a stronger imprint on current individual abundance than on species richness, our study highlights population‐level responses as a sensitive and early‐warning indicator of long‐term biodiversity erosion in agricultural landscapes. Our results therefore add a novel facet to the increasingly well‐established assessment of widespread insect declines (Seibold et al. 2019; Cardoso et al. 2020; Didham et al. 2020; Rumohr et al. 2023), underpinning that the temporal dimension in assessing the state of biodiversity is still severely underrated.

## Author Contributions

**Elisabeth Glatzhofer:** conceptualization (equal), data curation (lead), formal analysis (equal), methodology (equal), writing – original draft (lead). **Bernd Lenzner:** conceptualization (supporting), formal analysis (equal), methodology (equal), supervision (supporting), visualization (supporting), writing – review and editing (supporting). **Tobias Schernhammer:** conceptualization (equal), data curation (equal), formal analysis (equal), methodology (equal), supervision (supporting), writing – review and editing (supporting). **Franz Essl:** conceptualization (equal), formal analysis (equal), methodology (equal), supervision (lead), writing – review and editing (lead).

## Funding

This research was funded in whole or in part by the Austrian Science Fund (FWF) [10.55776/I6809 and 10.55776/I5825]. T.S. appreciates funding by the Austrian Science Foundation (FWF; pr.no. 35689‐B).

## Conflicts of Interest

The authors declare no conflicts of interest.

## Supporting information


**Table S1:** Sampled pastures within the study area. Code names of the pastures refer to those used in Figure 1. *Any use of anthelmintics, whether it is regular or only in case of worm infestation was treated as a “yes”.
**Table S2:** Species found in the study area with total number of counts, total number of occupied pastures by each species, nesting behavior, habitat preference, dung specialization, and population trend (see text for detailed explanation). Nesting behavior: e = endocoprid, p = paracoprid, t = telecoprid, * = phyto‐saprophagous species with development in detritus; habitat: o = open landscape, s‐o = semi‐open landscapes; dung specialization: number of different animal‐dung used as a food source (low numbers indicate higher specialization). **For all model calculations with declining and increasing species, these species were left out, as they historically were often subject to identification errors and as valid identification features were only published after 2000 (1–3) or showed a stable range (***).
**Table S3:** Total land cover area [ha] of the main land cover types in all sampled sites at different time points.
**Table S4:** Impact of anthelmintics on the different ecological groups compared to land use. Diagnostic model values of the Generalized Linear Mixed Models: significance levels: . = 0.05 < *p* < 0.10, * = 0.01 < *p* < 0.05, ** = 0.001 < *p* < 0.01, 2022anth/2022, models calibrated with anthelmintics and without; AICc, corrected Akaike information criterion; est., estimates. Test statistics for the models calibrated without anthelmintics (2022) are shown at Table S6.
**Table S5:** Maps and aerial photographs used for land cover digitization.
**Table S6:** Model results of Generalized Linear Mixed Models (GLMMs) analyzing the impact of historic and recent land use on different ecological groups of dung beetles. Diagnostic model values of the Generalized Linear Mixed Models: significance levels: . = 0.05 < *p* < 0.10, * = 0.01 < *p* < 0.05, ** = 0.001 < *p* < 0.01, est., estimates.
**Figure S1:** Autocorrelation Function plot calculated for the generalized linear land use models, showing that no spatial autocorrelation bias between the sample sites occurs.

## Data Availability

Relevant data and code are publicly available via this repository: https://doi.org/10.5281/zenodo.18449305.
