# Supplementary material for: Evidence of Pervasive Legacy of Land Use Change on Dung Beetles in Central European Grazed Grasslands
Source: Ecol Evol. 2026 Apr 29;16(5):e73571. doi: 10.1002/ece3.73571 (PMC13127227; doi:10.1002/ece3.73571)
Supplement: Supplementary file 1 — Table S1: Sampled pastures within the study area. Code names of the pastures refer to those used in Figure 1. *Any use of anthelmintics, whether it is regular or only in case of worm infestation was treated as a “yes”. Table S2: Species found in the study area with total number of counts, total number of occupied pastures by each species, nesting behavior, habitat preference, dung specialization, and population trend (see text for detailed explanation). Nesting behavior: e = endocoprid, p = paracoprid, t = telecoprid, * = phyto‐saprophagous species with development in detritus; habitat: o = open landscape, s‐o = semi‐open landscapes; dung specialization: number of different animal‐dung used as a food source (low numbers indicate higher specialization). **For all model calculations with declining and increasing species, these species were left out, as they historically were often subject to identification errors and as valid identification features were only published after 2000 (1–3) or showed a stable range (***). Table S3: Total land cover area [ha] of the main land cover types in all sampled sites at different time points. Table S4: Impact of anthelmintics on the different ecological groups compared to land use. Diagnostic model values of the Generalized Linear Mixed Models: significance levels: . = 0.05 < p < 0.10, * = 0.01 < p < 0.05, ** = 0.001 < p < 0.01, 2022anth/2022, models calibrated with anthelmintics and without; AICc, corrected Akaike information criterion; est., estimates. Test statistics for the models calibrated without anthelmintics (2022) are shown at Table S6. Table S5: Maps and aerial photographs used for land cover digitization. Table S6: Model results of Generalized Linear Mixed Models (GLMMs) analyzing the impact of historic and recent land use on different ecological groups of dung beetles. Diagnostic model values of the Generalized Linear Mixed Models: significance levels: . = 0.05 < p < 0.10, * = 0.01 < p < 0.05, ** = 0.001 < p < 0.01, est [file ECE3-16-e73571-s001.docx]

**Supplemental Information for:**

Evidence of pervasive legacy of land use change on dung beetles in central European grazed grasslands

Elisabeth Glatzhofer^1^, Bernd Lenzner^2^, Tobias Schernhammer^1,2^, Franz Essl^2^

^1^Vienna Institute for Nature Conservation and Analyses, Giessergasse 6/7, 1090 Vienna, Austria

^2^ Division of BioInvasions, Global Change & Macroecology, Department of Botany and Biodiversity Research, University of Vienna, Rennweg 14, 1030 Vienna, Austria

Table S1. Sampled pastures within the study area. Code names of the pastures refer to those used in Figure 1. *Any use of anthelmintics, whether it is regular or only in case of worm infestation was treated as a “yes”.

| code | grazing animal | sampling date | anthelmintics* |
| --- | --- | --- | --- |
| ALB | cattle | 22.06.2021 | YES |
| DON | cattle | 14.06.2022 | YES |
| ECK | cattle | 07.06.2022 | NO |
| EGG | cattle | 01.05.2019 | NO |
| FMG | cattle | 08.06.2020 | NO |
| GRA | cattle | 15.06.2022 | NO |
| HAV | horses | 12.05.2018 | NO |
| HOF | horses | 07.06.2022 | YES |
| HOH | cattle | 15.06.2022 | YES |
| KIR | cattle | 22.06.2021 | YES |
| LAA | cattle | 11.06.2020 | YES |
| LAN | cattle | 22.06.2021 | YES |
| LED | horses | 14.04.2018 | YES |
| LTG | cattle | 16.06.2021 | NO |
| MAN | cattle | 15.06.2022 | YES |
| MAR | horses | 28.06.2019 | NO |
| MAS | horses | 12.05.2018 | YES |
| MGN | cattle | 28.04.2020 | NO |
| MIT | cattle | 14.04.2018 | YES |
| NES | horses | 14.04.2018 | NO |
| NIC | cattle | 29.06.2022 | NO |
| OGG | cattle | 14.06.2022 | YES |
| PRZ | horses | 22.06.2021 | YES |
| PUR | cattle | 14.06.2022 | NO |
| RAB | cattle | 15.06.2022 | NO |
| SAS | cattle | 08.06.2020 | NO |
| SEE | cattle | 22.06.2021 | YES |
| SGR | cattle | 22.06.2021 | YES |
| SHW | cattle | 28.06.2022 | NO |
| SKA | cattle | 14.04.2018 | NO |
| SUL | cattle | 14.06.2022 | YES |
| UNT | cattle | 28.04.2020 | NO |
| WER | cattle | 08.06.2020 | NO |
| ZUR | cattle | 29.06.2022 | NO |

Table S2. Species found in the study area with total number of counts, total number of occupied pastures by each species, nesting behavior, habitat preference, dung specialization and population trend (see text for detailed explanation). Nesting behavior: e = endocoprid, p = paracoprid, t = telecoprid, * = phyto-saprophagous species with development in detritus; habitat: o = open landscape, s-o = semi-open landscapes; dung specialization: number of different animal-dung used as a food source (low numbers indicate higher specialization). **For all model calculations with declining and increasing species, these species were left out, as they historically were often subject to identification errors and as valid identification features were only published after 2000 (1–3) or showed a stable range (***).

| species | total counts | inhabited pastures | nesting behaviour | habitat | dung specialisation | trend |
| --- | --- | --- | --- | --- | --- | --- |
| Geotrupidae | | | | | | |
| *Geotrupes spiniger* (Marsham, 1802) | 9 | 2 | p | s-o | 5 | 0.71 |
| *Geotrupes stercorarius* (Linnaeus, 1758) | 1 | 1 | p | s-o | 6 | 0.50 |
| *Trypocopris vernalis* (Linnaeus, 1758) | 10 | 3 | p | s-o | 5 | 1.22 |
| Scarabaeidae | | | | | | |
| *Acanthobodilus immundus* (Creutzer, 1799) | 131 | 13 | e | o | 4 | 1.07 |
| *Acrossus depressus* (Kugelann, 1792) | 1 | 1 | e | s-o | 8 | 0.75 |
| *Acrossus luridus*  (Fabricius, 1775) | 17 | 5 | e | s-o | 7 | 0.61 |
| *Acrossus rufipes*  (Linnaeus, 1758) | 1 | 1 | e | s-o | 9 | 0.25 |
| *Agrilinus ater*  (Degeer, 1774) | 3 | 2 | e | s-o | 7 | 0.38 |
| *Aphodius pedellus* (Linnaeus, 1758) [sensu Wilson 2001] | 124 | 18 | e | s-o | 8 | 0.48 |
| *Biralus satellitius*  (Herbst, 1789) | 4 | 2 | e | s-o | 3 | 0.10 |
| *Bodilopsis rufus*  (Moll, 1782) | 108 | 14 | e | s-o | 8 | 2.20 |
| *Bodilus lugens*  (Creutzer, 1799) | 190 | 12 | e | o | 5 | 0.56 |
| *Calamosternus granarius* (Linnaeus, 1767) | 132 | 10 | e | s-o | 9 | 0.34 |
| *Chilothorax distinctus* (Müller, 1776) | 100 | 4 | e | s-o | 8 | 0.68 |
| *Colobopterus erraticus* (Linnaeus, 1758) | 1504 | 23 | p | s-o | 8 | 0.84 |
| *Coprimorphus scrutator* (Herbst, 1789) | 212 | 18 | e | s-o | 4 | 0.88 |
| *Copris lunaris*  (Linnaeus, 1758) | 26 | 8 | p | s-o | 6 | 0.30 |
| *Esymus pusillus*  (Herbst, 1789) | 368 | 20 | e | s-o | 7 | 1.50 |
| *Euoniticellus fulvus*  (Goeze, 1777) | 814 | 26 | p | o | 4 | 1.00*** |
| *Euorodalus coenosus* (Panzer, 1798) | 6 | 1 | e | s-o | 8 | 0.22** |
| *Euorodalus paracoenosus* (Balthasar & Hrubant, 1960) | 176 | 16 | e | o | 5 | 7.50** |
| *Eupleurus subterraneus* (Linnaeus, 1758) | 11 | 6 | e | s-o | 7 | 0.29 |
| *Labarrus lividus*  (Olivier, 1789) | 18 | 4 | e | s-o | 4 | 1.50 |
| *Limarus maculatus*  (Sturm, 1800) | 2 | 1 | e | s-o | 7 | 1.50 |
| *Melinopterus consputus* (Creutzer, 1799) | 1 | 1 | e | o | 6 | 1.57 |
| *Melinopterus prodromus* (Brahm, 1790) | 80 | 3 | e | s-o | 9 | 0.58 |
| *Melinopterus sphacelatus* (Panzer, 1798) | 94 | 2 | e | s-o | 8 | 0.57 |
| *Nialus varians* (Duftschmid, 1805) | 5 | 2 | e | o | 3 | 0.44 |
| *Onthophagus coenobita* (Herbst, 1783) | 64 | 11 | p | s-o | 8 | 0.85 |
| *Onthophagus fracticornis* (Preyssler, 1790) | 49 | 9 | p | s-o | 8 | 0.86 |
| *Onthophagus furcatus* (Fabricius, 1781) | 69 | 5 | p | o | 6 | 0.39 |
| *Onthophagus illyricus* (Scopoli, 1763) | 881 | 22 | p | s-o | 5 | 23.00** |
| *Onthophagus joannae* Goljan, 1953 | 118 | 9 | p | s-o | 9 | 2.00** |
| *Onthophagus lemur* (Fabricius, 1781) | 37 | 2 | p | o | 7 | 0.25 |
| *Onthophagus medius* (Kugelann, 1792) | 3 | 3 | p | o | 3 | 0.57 |
| *Onthophagus nuchicornis* (Linnaeus, 1758) | 66 | 8 | p | s-o | 8 | 0.60 |
| *Onthophagus ovatus* (Linnaeus, 1767) | 513 | 22 | p | s-o | 8 | 1.00** |
| *Onthophagus ruficapillus* Brullé, 1832 | 2130 | 23 | p | o | 2 | 0.80 |
| *Onthophagus taurus* (Schreber, 1759) | 429 | 20 | p | s-o | 6 | 1.09** |
| *Onthophagus vacca* (Linnaeus, 1767) | 46 | 14 | p | o | 3 | 0.69 |
| *Onthophagus verticicornis* (Laicharting, 1781) | 211 | 9 | p | s-o | 8 | 0.39 |
| *Onthophagus vitulus* (Fabricius, 1776) | 3 | 2 | p | o | 7 | 0.11 |
| *Otophorus haemorrhoidalis*  (Linnaeus, 1758) | 341 | 26 | e | s-o | 8 | 1.80 |
| *Oxyomus sylvestris* (Scopoli, 1763) | 11 | 5 | * | s-o | 4 | 2.00 |
| *Phalacronothus biguttatus* (Germar, 1823) | 2 | 2 | e | s-o | 7 | 0.40 |
| *Plagiogonus arenarius* (Olivier, 1789) | 4 | 4 | e | o | 6 | 0.15 |
| *Planolinus borealis* Gyllenhal, 1827 | 1 | 1 | e | s-o | 5 | 1.00*** |
| *Pleurophorus caesus* (Panzer, 1796) | 2 | 2 | * | o | 3 | 0.11 |
| *Pleurophorus pannonicus* Petrovitz, 1961 | 2 | 2 | * | o | 3 | 0.60 |
| *Rhodaphodius foetens* (Fabricius, 1787) | 30 | 7 | e | s-o | 4 | 1.57 |
| *Sigorus porcus*  (Fabricius, 1792) | 1 | 1 | e | s-o | 6 | 1.20 |
| *Sisyphus schaefferi* (Linnaeus, 1758) | 22 | 3 | t | s-o | 7 | 0.95 |
| *Subrinus sturmi*  (Harold, 1870) | 433 | 9 | e | o | 3 | 3.50 |
| *Teuchestes fossor* (Linnaeus, 1758) | 91 | 13 | e | s-o | 6 | 0.25 |
| *Trichonotulus scrofa* (Fabricius, 1787) | 15 | 5 | e | s-o | 7 | 0.44 |
| *Volinus sticticus*  (Panzer, 1798) | 16 | 8 | e | s-o | 7 | 0.90 |
| total: 56 | **9738** |  |  |  |  |  |

Table S3. Total land cover area [ha] of the main land cover types in all sampled sites at different time points.

|  | land cover types [ha] | | | |
| --- | --- | --- | --- | --- |
| year | **fields** | **settlements** | **forests** | **pastures** |
| 1900 | 4477 | 211 | 1424 | 2293 |
| 1950 | 5901 | 352 | 1471 | 1177 |
| 1990 | 5649 | 501 | 1892 | 821 |
| 2022 | 4875 | 614 | 2101 | 1218 |

Table S4. Impact of anthelmintics on the different ecological groups compared to land use. Diagnostic model values of the Generalized Linear Mixed Models: significance levels: . = 0.05<p<0.10, * = 0.01<p<0.05, ** = 0.001<p<0.01, est = estimates, AICc = corrected Akaike information criterion, 2022anth/ 2022 = models calibrated with anthelmintics and without. Test statistics for the models calibrated without anthelmintics (2022) are shown at Table S6.

|  | **2022anth** | | **AICc** | | **R^2^** | |
| --- | --- | --- | --- | --- | --- | --- |
| **ecological group** | **p-values** | **est** | **2022anth** | **2022** | **2022anth** | **2022** |
| **all** | **0.01 **** | -0.309 | 228.04 | 231.70 | 0.35 | 0.18 |
| **open landscape** | **0.06 .** | -0.41 | 175.67 | 175.78 | 0.50 | 0.41 |
| **semi-open landscape** | **0.03 *** | -0.30 | 194.25 | 188.63 | 0.30 | 0.19 |
| **paracoprid** | 0.41 | -0.13 | 174.97 | 172.15 | 0.21 | 0.19 |
| **endocoprid** | **0.01 *** | -0.42 | 190.30 | 193.11 | 0.29 | 0.08 |
| **declining** | **0.01 *** | -0.38 | 187.11 | 189.89 | 0.27 | 0.16 |
| **increasing** | **0.03 *** | -0.55 | 147.95 | 149.18 | 0.41 | 0.29 |

Table S5. Maps and aerial photographs used for land cover digitization.

| **time period** | **digitized map** | **data source** |
| --- | --- | --- |
| **1880-1900** | administrative map of Lower Austria (1867-1882) | Office of the State Government of Lower Austria, 2022 |
|  | “Third Military Survey” (Franzisco-Josephinische Landesaufnahme; 1880s) | Austrian Federal Office of Metrology and Surveying |
| **1950-1960** | aerial photographs | Austrian Federal Office of Metrology and Surveying |
| **1990-1995** | aerial photographs | Austrian Federal Office of Metrology and Surveying |
| **2020-2022** | satellite images | Google Maps, 2022 |

Table S6. Model results of Generalized Linear Mixed Models (GLMMs) analyzing the impact of historic and recent land use on different ecological groups of dung beetles. Diagnostic model values of the Generalized Linear Mixed Models: significance levels: . = 0.05<p<0.10, * = 0.01<p<0.05, ** = 0.001<p<0.01, est = estimates.

|  |  |  | 2022 | | 1990 | | 1950 | | 1880 | |
| --- | --- | --- | --- | --- | --- | --- | --- | --- | --- | --- |
|  |  | ecological group | p-values | est | p-values | est | p-values | est | p-values | est |
| SPECIES RICHNESS | SETTL | all | 0.07 . | 0.09 | 0.05 . | 0.10 | 0.19 | 0.07 | 0.48 | -0.03 |
|  |  | open landscape | 0.38 | 0.08 | 0.38 | 0.08 | 0.84 | 0.02 | 0.37 | -0.09 |
|  |  | semi-open landscape | 0.07 . | 0.09 | 0.06 . | 0.10 | 0.10 | 0.09 | 0.89 | -0.01 |
|  |  | paracoprid | 0.00 ** | 0.06 | 0.01 ** | 0.17 | 0.08 . | 0.13 | 0.50 | 0.04 |
|  |  | endocoprid | 0.84 | 0.01 | 0.56 | 0.04 | 0.70 | 0.03 | 0.13 | -0.11 |
|  |  | declining | 0.03 * | 0.13 | 0.03 * | 0.14 | 0.09 . | 0.11 | 0.72 | -0.02 |
|  |  | increasing | 0.60 | -0.05 | 0.799 | -0.03 | 0.83 | -0.02 | 0.30 | -0.11 |
|  | FOREST | all | 0.71 | 0.02 | 0.08 . | 0.12 | 0.12 | 0.10 | 0.01 * | 0.15 |
|  |  | open landscape | 0.04 * | -0.25 | 0.57 | -0.08 | 0.41 | -0.10 | 0.96 | -0.01 |
|  |  | semi-open landscape | 0.17 | 0.10 | 0.04 * | 0.17 | 0.02 * | 0.17 | 0.01 ** | 0.20 |
|  |  | paracoprid | 0.96 | 0.09 | 0.18 | 0.14 | 0.47 | 0.07 | 0.04 * | 0.19 |
|  |  | endocoprid | 0.97 | 0.00 | 0.57 | 0.06 | 0.46 | 0.07 | 0.40 | 0.07 |
|  |  | declining | 0.80 | -0.02 | 0.26 | 0.11 | 0.14 | 0.13 | 0.01 * | 0.20 |
|  |  | increasing | 0.75 | 0.04 | 0.71 | 0.05 | 0.90 | 0.02 | 0.91 | 0.01 |
|  | AGRI | all | 0.76 | -0.02 | 0.11 | 0.12 | 0.24 | 0.08 | 0.07 . | 0.13 |
|  |  | open landscape | 0.20 | -0.14 | 0.35 | 0.13 | 0.83 | 0.03 | 0.44 | 0.09 |
|  |  | semi-open landscape | 0.76 | 0.02 | 0.21 | 0.12 | 0.17 | 0.11 | 0.11 | 0.12 |
|  |  | paracoprid | 0.68 | -0.04 | 0.26 | 0.13 | 0.46 | 0.07 | 0.04 * | 0.21 |
|  |  | endocoprid | 0.97 | 0.00 | 0.48 | 0.08 | 0.72 | 0.04 | 0.98 | 0.00 |
|  |  | declining | 0.44 | -0.06 | 0.30 | 0.11 | 0.19 | 0.12 | 0.09 . | 0.15 |
|  |  | increasing | 0.95 | 0.01 | 0.73 | 0.05 | 0.73 | -0.05 | 0.69 | -0.06 |
|  | PAST | all | 0.82 | -0.01 | 0.61 | 0.03 | 0.74 | -0.02 | 0.55 | -0.04 |
|  |  | open landscape | 0.41 | 0.07 | 0.41 | 0.09 | 0.74 | -0.04 | 0.83 | 0.02 |
|  |  | semi-open landscape | 0.35 | -0.06 | 0.82 | -0.02 | 0.66 | -0.03 | 0.21 | -0.09 |
|  |  | paracoprid | 0.55 | 0.07 | 0.46 | 0.06 | 0.98 | 0.00 | 0.43 | 0.07 |
|  |  | endocoprid | 0.31 | -0.08 | 0.95 | 0.01 | 0.95 | -0.01 | 0.15 | -0.13 |
|  |  | declining | 0.56 | -0.04 | 0.97 | 0.00 | 0.57 | -0.05 | 0.71 | -0.03 |
|  |  | increasing | 0.94 | 0.01 | 0.90 | 0.02 | 0.96 | -0.01 | 0.31 | -0.12 |
| INDIVIDUAL ABUNDANCE | SETTL | all | 0.29 | -0.09 | 0.13 | -0.13 | 0.16 | -0.13 | <2e-16 *** | -0.25 |
|  |  | open landscape | 0.20 | -0.21 | 0.26 | -0.19 | 0.25 | -0.20 | 0.36 | -0.16 |
|  |  | semi-open landscape | 0.62 | -0.05 | 0.25 | -0.12 | 0.29 | -0.11 | 0.00 ** | -0.27 |
|  |  | paracoprid | 0.99 | 0.00 | 0.94 | -0.01 | 0.88 | -0.02 | 0.054 . | -0.25 |
|  |  | endocoprid | 0.03 * | -0.23 | 0.01 ** | -0.30 | 0.02 * | -0.27 | 0.00 ** | -0.35 |
|  |  | declining | 0.56 | -0.06 | 0.62 | -0.06 | 0.27 | -0.13 | 0.03 * | -0.23 |
|  |  | increasing | 0.15 | -0.24 | 0.11 | -0.27 | 0.26 | -0.19 | 0.01 ** | -0.43 |
|  | FOREST | all | 0.16 | 0.13 | 0.32 | 0.12 | 0.48 | 0.08 | <2e-16 *** | 0.16 |
|  |  | open landscape | 0.88 | -0.02 | 0.95 | 0.01 | 0.84 | -0.04 | 0.99 | 0.00 |
|  |  | semi-open landscape | 0.038 * | 0.22 | 0.16 | 0.20 | 0.19 | 0.18 | 0.02 * | 0.27 |
|  |  | paracoprid | 0.41 | 0.12 | 0.41 | 0.16 | 0.72 | 0.07 | 0.17 | 0.23 |
|  |  | endocoprid | 0.24 | 0.13 | 0.56 | 0.08 | 0.34 | 0.13 | 0.48 | 0.09 |
|  |  | declining | 0.37 | -0.12 | 0.37 | -0.14 | 0.49 | -0.10 | 0.80 | 0.04 |
|  |  | increasing | 0.19 | 0.20 | 0.53 | 0.13 | 0.34 | 0.18 | 0.31 | 0.18 |
|  | AGRI | all | 0.39 | 0.08 | 0.96 | 0.01 | 0.78 | -0.03 | <2e-16 *** | 0.05 |
|  |  | open landscape | 0.54 | 0.10 | 0.70 | 0.09 | 0.82 | -0.05 | 0.66 | 0.09 |
|  |  | semi-open landscape | 0.34 | 0.10 | 1.00 | 0.00 | 0.87 | 0.02 | 0.37 | 0.10 |
|  |  | paracoprid | 0.20 | 0.18 | 0.51 | 0.14 | 0.86 | -0.03 | 0.52 | 0.10 |
|  |  | endocoprid | 0.83 | -0.02 | 0.47 | -0.12 | 0.96 | 0.01 | 0.84 | -0.03 |
|  |  | declining | 0.99 | 0.00 | 0.92 | 0.02 | 0.49 | -0.10 | 0.72 | 0.05 |
|  |  | increasing | 0.34 | -0.15 | 0.37 | -0.20 | 0.68 | -0.08 | 0.39 | -0.15 |
|  | PAST | all | 0.61 | 0.04 | 0.76 | 0.03 | 0.85 | 0.02 | <2e-16 *** | 0.02 |
|  |  | open landscape | 0.68 | -0.06 | 0.78 | 0.04 | 0.88 | 0.02 | 0.83 | 0.04 |
|  |  | semi-open landscape | 0.51 | 0.07 | 0.88 | -0.02 | 0.86 | -0.02 | 0.84 | 0.02 |
|  |  | paracoprid | 0.79 | 0.04 | 0.49 | 0.11 | 0.45 | 0.11 | 0.75 | 0.05 |
|  |  | endocoprid | 0.75 | 0.03 | 0.51 | -0.08 | 0.54 | -0.07 | 0.56 | -0.07 |
|  |  | declining | 0.66 | 0.05 | 0.34 | 0.12 | 0.96 | -0.01 | 0.71 | 0.05 |
|  |  | increasing | 0.80 | 0.04 | 0.63 | -0.09 | 0.61 | -0.08 | 0.26 | -0.17 |

Figure S1. Autocorrelation Function plot calculated for the generalized linear land use models, showing that no spatial autocorrelation bias between the sample sites occurs.


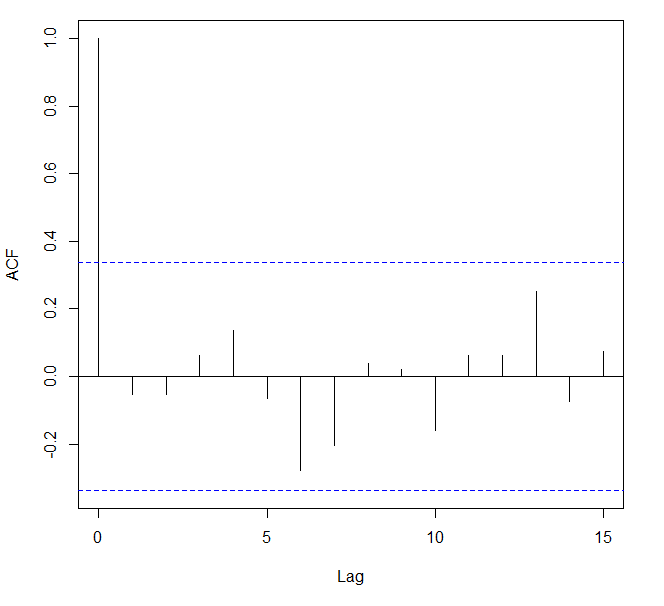


**SI References**

1. E. Rößner, Die Verbreitung von Aphodius (Euorodalus) coenosus (PANZER, 1798) und Aphodius (Euorodalus) paracoenosus BALTHASAR & HRUBANT, 1960 in Deutschland und Mitteilung von Funddaten zu den Gesamtarealen beider Arten (Coleoptera: Scarabaeidae). *Entomologische Zeitschrift* **114** (2004).

2. E. Rößner, Ein weiteres differenzialdiagnostisches Merkmal zur Unterscheidung zwischen Onthophagus ovatus (Linnaeus, 1767) und O. joannae Goljan, 1953 (Coleoptera, Scarabaeidae). *Virgo* **9**, 30–32 (2006).

3. A. Pizzo, *et al.*, Genetic and morphological differentiation patterns between sister species: the case of Onthophagus taurus and Onthophagus illyricus (Coleoptera, Scarabaeidae). *Biological Journal of The Linnean Society* **89**, 197–211 (2006).
